# Occurrences of non-linear phenomena and vocal harshness in dog whines as indicators of stress and ageing

**DOI:** 10.1038/s41598-021-83614-1

**Published:** 2021-02-24

**Authors:** András Marx, Rita Lenkei, Paula Pérez Fraga, Viktória Bakos, Enikő Kubinyi, Tamás Faragó

**Affiliations:** 1 Department of Ethology, Eötvös Loránd University, Budapest, Hungary; 2 MTA-ELTE ‘Lendület’ Neuroethology of Communication Research Group, Eötvös Loránd Research Network, Budapest, Hungary

**Keywords:** Animal behaviour, Behavioural methods, Emotion, Social behaviour, Ageing

## Abstract

During social interactions, acoustic parameters of tetrapods’ vocalisations reflect the emotional state of the caller. Higher levels of spectral noise and the occurrence of irregularities (non-linear phenomena NLP) might be negative arousal indicators in alarm calls, although less is known about other distress vocalisations. Family dogs experience different levels of stress during separation from their owner and may vocalise extensively. Analysing their whines can provide evidence for the relationship between arousal and NLP. We recorded 167 family dogs’ separation behaviour including vocalisations, assessed their stress level based on behaviour and tested how these, their individual features, and owner reported separation-related problems (SRP) relate to their whines’ (N = 4086) spectral noise and NLP. Dogs with SRP produced NLP whines more likely. More active dogs and dogs that tried to escape produced noisier whines. Older dogs’ whines were more harmonic than younger ones’, but they also showed a higher NLP ratio. Our results show that vocal harshness and NLP are associated with arousal in contact calls, and thus might function as stress indicators. The higher occurrence of NLP in older dogs irrespective to separation stress suggests loss in precise neural control of the larynx, and hence can be a potential ageing indicator.

## Introduction

It is now widely accepted that expressing emotions is one of the main communicative functions of animal vocalisations. When trying to explain the link between inner states and the acoustic features of vocalisations, Morton formulated his motivational–structural rules based on morphological effects on call structure^1^. He suggested that size constrains, causing larger individuals to have lower pitch and smaller ones higher through a ritualization process, which led to the association between dominance and low pitch and submission with high pitch. However, this cannot explain the evolution of the full spectrum of possible inner state associated vocalisations. The Source-Filter framework^2,3^ proposes the division of the vocal apparatus into two functional parts with their related acoustic effect on the vocal result: firstly the source—the lungs and the larynx (affecting mainly pitch related parameters), and secondly the filter—the cavities and obstacles between the larynx and the nose or mouth (modifying the spectral structure). These two together but independently form the vocal output during communication. Thanks to this approach, we have gained a better understanding of how neural changes due to emotions affect different aspects of sound production, resulting in different vocal outputs that provide the basis of general emotion encoding rules^4,5^. Simply put, the arousal (low or high emotional intensity) and valence (positive or negative emotional load) state of an individual affects the muscles participating in vocal production, resulting in different tension states of the vocal folds and the muscles involved in sound production leading to specific vocal structure, through which listeners are able to assess the emotional state of the caller.

While the role of pitch in arousal communication is studied in a wide range of species^5^, one other source related but more enigmatic peculiarity of sound production, the occurrence of so-called non-linear phenomena (NLP) is less understood. NLP are irregularities, involving abrupt changes in the harmonic structure of sound produced in the larynx, caused by the asynchronization of the two vocal folds’ oscillations^6^. The occurrence of NLP is due to the fact that the two vocal folds act as coupled oscillators, and each vocal fold movement affects the other’s, thus even small differences between their movements can lead to complex vibration patterns and abrupt transitions between periodic, quasiperiodic and non-periodic vibratory states^7^.

**Figure 1:**
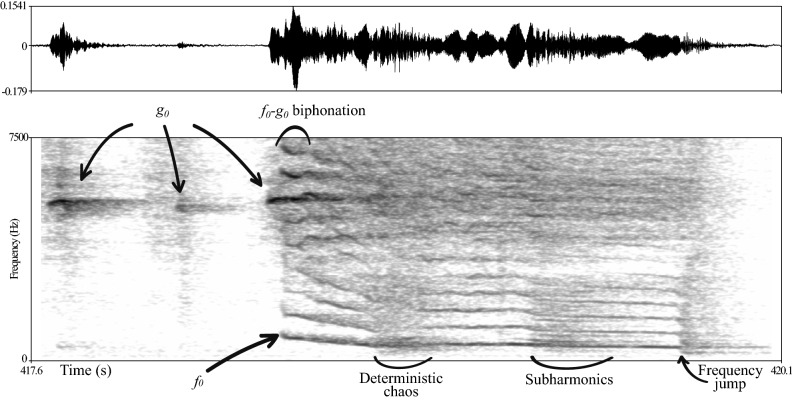
One example of a dog whine containing several types of non-linear phenomena. *f*_0_ label identifies whines with the lower fundamental frequency, while *g*_0_ the high secondary fundamental frequency (whistle whine or squeak in other nomenclatures). The co-occurrence of these two marked as biphonation which was not counted as NLP in the current analysis, while other type of NLP (deterministic chaos; subharmonics; frequency jump) were included. (The figure is the work of the first author).

Different types of NLP can be found in vocalisations^6,8,9^ (Fig. 1), such as the appearance of quasiperiodic vibrations called *subharmonics* when the vocal folds start to move with different frequencies. The folds stay partly in synchrony due to their coupling, as the periodicity of the vibration of one vocal fold is an integer fraction of the other (e.g. 1:2, 2:3). Asynchrony of the vocal folds can also occur, causing sudden loss of harmonic structure and appearance of harshness called *deterministic chaos*. *Frequency jumps* occur when the vocal folds remain in synchrony, but reach a different vibratory state without transition^1^. Finally, *biphonation* can occur when two independent (e.g. an additional source in the vocal tract^10^) or seemingly independent (e.g. vocal lips on the vocal folds^11^) sound sources function in parallel producing two fundamental frequencies (*f*_0_ and *g*_0_) during vocalisation, or due to the modulation of the fundamental frequency (*f*_0_) by lower frequency vibrations^12^. These phenomena can be differentiated from subharmonics due to the fact that the ratio between the two pitches is not a fraction of integers.

Initially these irregularities were considered as abnormalities of voice production, but research revealed that they are inherent in the sound production mechanisms and common in normal vocalisations of tetrapods^9^. While it is possible that NLP are just by-products, Fitch and colleagues^8^ argued that natural selection can exploit their occurrences, e.g., promoting individual recognition, helping the transmission of indexical cues or providing information of health or genetic quality. Blumstein & Récapet^13^ suggested that as nonlinearities in the sound are more prominent when the vocal apparatus is in extremely tensed state, due to e.g. stress, they are reflective of the callers’ arousal. While a few studies have collected data supporting the adaptive functions of NLP in alarm calls (e.g. unpredictability:^14,15^; arousal:^16^) little is known about other distress vocalisations.

In distress vocalisations such as separation calls NLP are quite prominent^17^ (for an example see Fig. 1). Such arousal level cues might have adaptive significance, as these calls are used in contexts when the caller is in need and dependent on another individual or a social group^18^. Several studies found a positive relationship between spectral noise and arousal in contact calls (e.g., marmosets^19^, goats^20^, piglets^21^) but none attempted to find associations with the occurrence of NLP.

Family dogs’ whines emitted in separation from the owner, are high-pitched and generally tonal but are rich in NLP^22,23^. They can be considered as contact calls functioning to evoke the attention of the owner^24^, and they possibly developed from the pup whine, which originally functioned as a separation call in the absence of the mother^25^. NLP have already been described in Canid vocalisations (dhole:^26^; red wolf:^22^), including dogs’ howls^11^, barks^27^ and whines^23^, but none of these studies have directly tested what role they play in communication.

We can assume that the occurrence of nonlinearities^28^ and other common measures of spectral noise (harmonic-to-noise ratio [HNR], jitter and wiener entropy) are honest cues of negative arousal^4,29^, and thus may play a role in the communication of distress in dogs. We analysed whines from family dogs displaying different levels of stress during separation from their owner in an unfamiliar environment. Although high frequency whistle whines (or *g*_0_ whines) are a prominent part of the canid vocal repertoire^30^ and they are very common in dogs too^31^, we excluded them from the current analysis as they can form biphonations with the ‘regular’ *f*_0_ whines. Our reasoning for this was based on the findings of Frey et al. (2016)^10^ suggesting that their production most probably happens in the nasal cavities based on turbulent flow, and they are thus independent from the state and movements of the vocal folds. Therefore *g*_0_ whines are far less variable than *f*_0_ whines and supposedly play a lesser role in dynamic emotion communication (but probably act as individual^26^ and indexical cues^31^). Based on behavioural data we formed scales using principle component analysis (PCA) and used these as covariates, along with age, reproductive and owner-reported separation related problems (SRP) status and life history to identify their tonality and NLP correlates in whines. The PCA identified five different behavioural scales (for details see Table S3):

1. **chair/move**: moving, panting, orientating to the chair and exploring it (standardized Cronbach α = 0.636)
2. **escape**: rearing, jumping up and scratching the door and the wall, barking and yelping (standardized Cronbach α = 0.712)
3. **chair proximity**: staying close to the chair and far from the door (standardized Cronbach α = 0.577)
4. **tail-wagging/other vocalisations**: tail wagging, using other vocalisations and orienting to the door (standardized Cronbach α = 0.411)
5. **sit**: sitting and not standing (standardized Cronbach α = 0.698)

We expected that in more aroused dogs (characterised by increased activity, barking, yelping and other vocalisations and/or escape attempts) we would find a higher ratio of whines with NLP (higher NLP ratio) and overall a lower tonality (lower HNR, higher jitter and entropy) of the calls. We can also assume that dogs that are struggling with separation related problems (SRP) according to their owner, experience higher level of stress, and thus produce noisier whines with more NLP. Additionally, dogs’ individual features (sex and reproductive status due to hormonal effects) and life history (previous experience of traumatic event/s) can also affect their stress level, thus we expected that neutered dogs and dogs with traumatic life events will produce harsher whines with more NLP, as they experience separation as a more stressful situation^32,33^. Finally, ageing can have different effects on the expression of NLP: in older dogs we can expect higher noise and more NLP due to the loss of neural control and/or degradation of tissues in the vocal apparatus, or alternatively due to higher experienced stress^34^. On the contrary, changes in the central nervous system might lead to lower stress in separation (positivity effect^35^) and thus higher tonality and fewer NLP.

## Results and discussion

From the 167 dogs 139 individuals produced whines during the separation. From these whining dogs 121 emitted *f*_0_ whines (number of whines: 4086; individual mean ± SD: 29.14 ± 36.89), and 90 individuals had at least one NLP in their whines. We found that dogs with owner-reported separation problems emitted these NLP whines with a higher chance (BinGLM: odds r. = 3.237; β ± SE = 1.175 ± 0.419; z = 2.802; *p* = 0.005) than dogs with no SRP. This suggests that individual separation stress level might be associated with NLP. We can assume that the presence of NLP in these dogs’ separation whines might affect the perception of their stress level by the owner in everyday contexts. NLP have a strong attention evoking and maintaining effect in alarm calls^13–15^, this might be present in separation and contact calls too, making them more salient. Thus, owners might be more aware of these dogs’ separation behaviour and might be more likely to report SRP in them. Additionally, recent results based on resynthesized human emotion expressions shown that addition of NLP to these calls raised the perception of negative valence which effect might be also a driving force behind owners’ higher awareness of their dogs’ negative state during separation.

**Figure 2:**
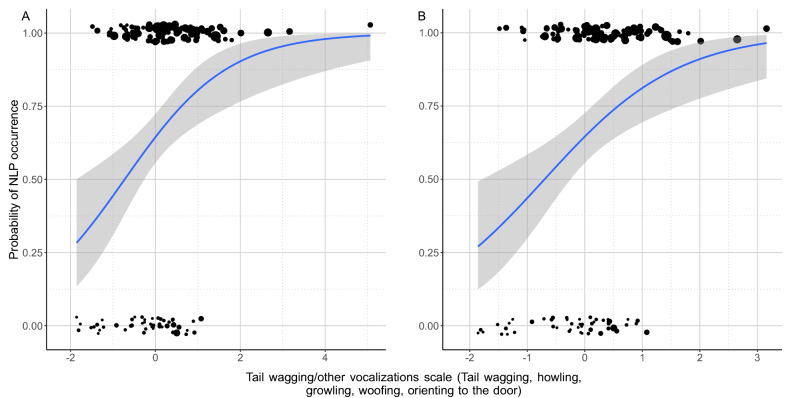
The positive association between probability to produce NLP whines among dogs and the time they spent with tail wagging, other vocalizations and orienting to the door. (**A**) The relationship shown in the original dataset, (**B**) the relationship shown after excluding the outliers. The size of the dots is proportional with the number of whines produced. The grey field shows the 95% confidence interval of the logistic fit.

We also found that dogs that vocalised, wagged their tail and oriented to the door for longer (higher PC4 scores) had a higher chance to have NLP in their whines (BinGLM: odds r. = 2.353; β ± SE = 0.856 ± 0.250; z = 3.421; *p* = 0.001; Fig. 2A,B).

A higher level of tail-wagging and door orientation, associated with other vocalisations including growling, woofing and howling might indicate a mixed negative inner state in dogs (although we have to note the low internal consistency [α = 0.411] in this component suggesting higher individual variance in these behaviours thus a lower reliability of this scale).

**Figure 3:**
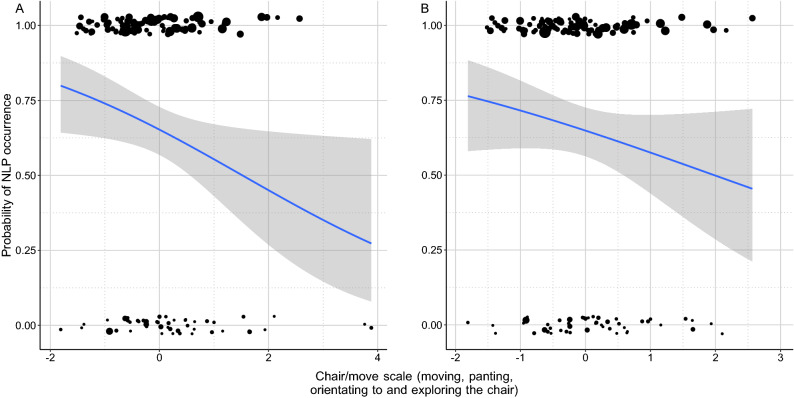
The negative association between probability to produce NLP whines among dogs and the time they spent with moving panting and chair directed behaviours. (**A**) The relationship shown in the original dataset, (**B**) The relationship shown after excluding the outliers. The size of the dots is proportional with the number of whines produced. The grey field shows the 95% confidence interval of the logistic fit.

Dogs that moved more (higher PC1 scores) had a lower chance to have NLP in their whines (BinGLM: odds r. = 0.641; β ± SE =  − 0.445 ± 0.205; z =  − 2.618; *p* = 0.030; Fig. 3A), although this effect was probably present only due to two influential datapoints (BinGLM after excluding extreme values: odds r. = 0.687; β ± SE =  − 0.358 ± 0.230; z =  − 1.576; *p* = 0.115; Fig. 3B). These two dogs spent most of their time during the separation alternating between restless circling in the room and standing close to the chair looking and sniffing at it. In any other regards their behaviour was not extreme.

**Figure 4:**
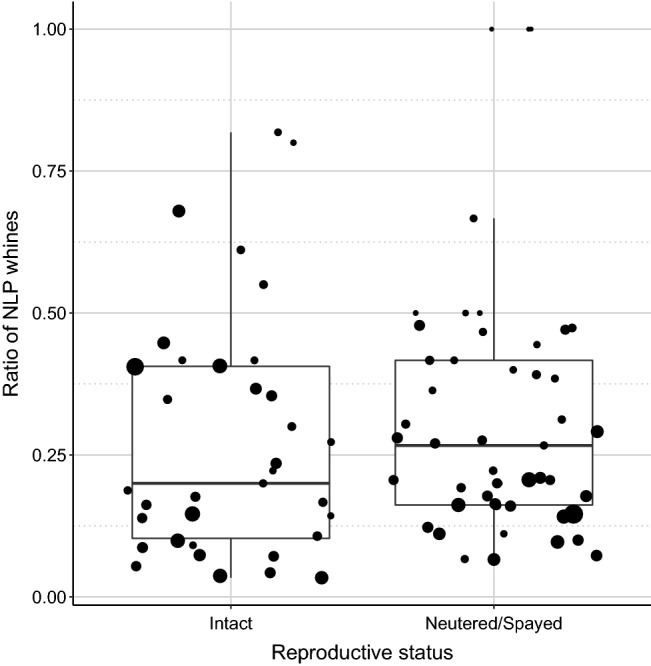
The effect of reproductive status on NLP ratio. Neutered/spayed dogs produce more whines containing NLP. The horizontal line within the box shows the median, the boxes represent the interquartile intervals, the whiskers the range. The size of the dots is proportional with the number of whines produced.

Reproductive status had a two-fold effect. On one hand intact dogs produced NLP whines with a higher chance (BinGLM: odds r. = 0.422; β ± SE =  − 0.863 ± 0.427; z =  − 2.019; *p* = 0.043), but in contrast the NLP ratio was higher in neutered/spayed dogs (QBin GLM: β ± SE =  − 0.400 ± 0.198; z =  − 2.021; *p* = 0.047; Fig. 4). This might seem contradictory. A higher prevalence of NLP producing individuals among intact dogs itself is unexpected, as based on the literature one would expect that gonadectomy co-occurs with SRP^36,37^. Among rescued and shelter dogs there is a higher prevalence of separation problems and these dogs are routinely spayed/neutered^38^, also owners might see this procedure as a solution for problematic behaviours. However, McGreevy and Masters (2008) found a higher prevalence of SRP in intact dogs^39^, thus it is still possible that our finding is due to a higher stress level of intact dogs in comparison to neutered dogs. In contrast, the higher ratio of NLP in neutered/spayed dogs might be due to physiological reasons. Low levels of gonadal and other sexual hormones might affect vocal production through their effect on mucous tissues^40^. In the lack of these hormones, the vocal folds are expected to have drier epithelial cover, promoting spectral noise and occurrences of NLP^41^. In line with these, in our sample gonadectomized individuals had a more variable overall whine structure.

**Figure 5:**
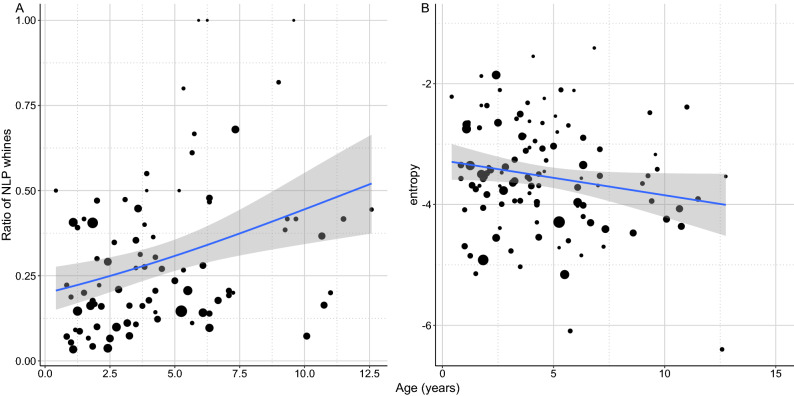
(**A**) The positive relationship between the ratio of NLP whines produced by the dog during the separation test and the dog’s age. (**B**) The negative relationship between the entropy of whines produced by the dog during the separation test and the dog’s age. The size of the dots is proportional with the number of whines produced. The grey field shows the 95% confidence interval of the linear fit.

Age had a positive association with the ratio of NLP whines (QBin GLM: β ± SE = 0.087 ± 0.036; t = 2.405; *p* = 0.018; Fig. 5A). Older dogs had more whines containing NLP than young ones. In contrast, wiener entropy was lower in older dogs (LM: β =  − 0.071; t =  − 2.302; *p* = 0.023, Fig. 5B).

These two findings are again seemingly in contradiction, but a higher number of NLP does not necessarily mean higher entropy on average. As NLP have sudden on- and offsets within one vocalisation and do not affect the entire call, the overall measurement of the whole whine can still show low entropy, regardless of the presence of e.g., a frequency jump. The higher occurrence of NLP in older dogs can be the result of age-related decay of the vocal apparatus (loss of elastic fibres, changes of the epithelium, muscle atrophy, etc.) which has already been described in humans^41–44^. However, this would lead to rising vocal harshness, which was not the case. Alternatively, older dogs may experience elevated stress during separation, leading to a higher NLP ratio, but this should be reflected in harsher whines. On the other hand, the positivity effect^35^ suggesting lower separation stress in older dogs might explain the more tonal whines here. The most plausible explanation seems to be that the higher occurrence of NLP is associated with an independent process, like losing the precise neural control of the larynx.

**Figure 6:**
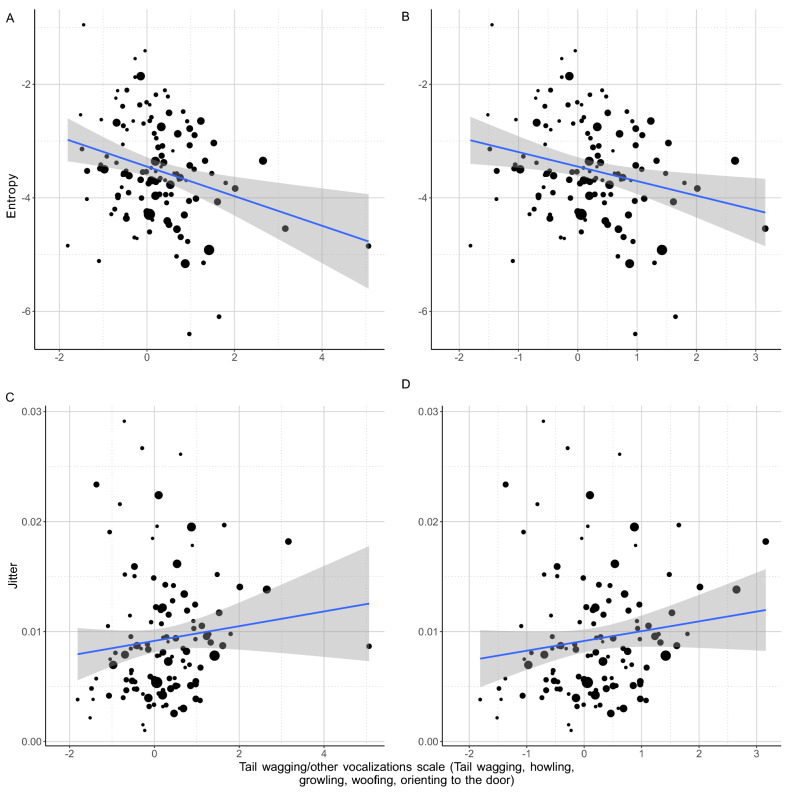
(**A**, **B**) The negative relationship between the entropy of whines produced by the dog during the separation test and the amount of time spent with tail wagging, other vocalizations and orienting to the door. (**C**, **D**) The positive relationship between the jitter of whines produced by the dog during the separation test and the amount of time spent with tail wagging, other vocalizations and orienting to the door. The size of the dots is proportional with the number of whines produced. The grey field shows the 95% confidence interval of the linear fit.

Dogs that vocalised, wagged their tail and oriented to the door for longer (higher PC4 scores) also emitted more harmonic whines (lower wiener entropy; LM: β =  − 0.238; t =  − 2.88; *p* = 0.005; Fig. 6A,B) but with slightly less stable *f*_0_ (higher jitter; LM: β = 0.008; t = 1.92; *p* = 0.057; without the influential point: β = 0.010; t = 2.116; *p* = 0.034; Fig. 6C,D).

Higher jitter indicates less regular vocal cycles leading to unstable *f*_0_, while lower entropy results in generally more tonal whines. It is possible that dogs wagging their tail, orienting to the door and producing various vocalisations were in a mixed inner state like frustration (a mix of rage and fear), leading to more variable vocal fold movements without a rise in spectral noise.

**Figure 7:**
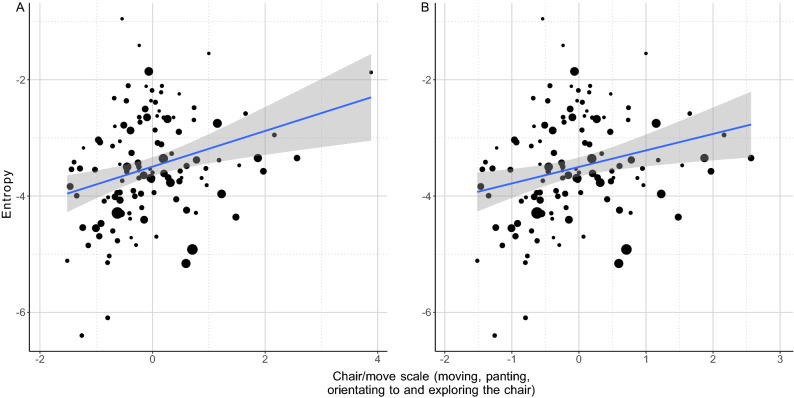
The positive relationship between the entropy of whines produced by the dog during the separation test and the amount of time spent with moving panting and chair directed behaviours. The size of the dots is proportional with the number of whines produced. The grey field shows the 95% confidence interval of the linear fit.

In the case of the chair/move scale (PC1) we found a positive association with wiener entropy (LM: β = 0.283; t = 3.11; *p* = 0.002; Fig. 7A,B): more active dogs spent more time orienting to and exploring the chair where the owner sat, and produced whines with a wider spectrum (higher entropy).

**Figure 8:**
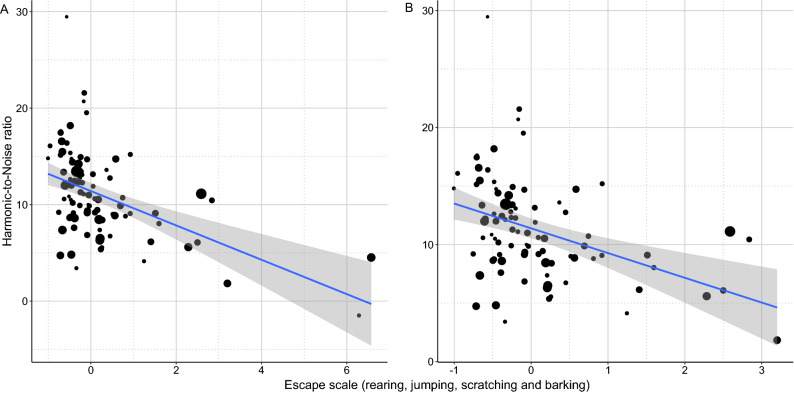
Figure The negative relationship between HNR and the dog’s escape activity during the separation test. The size of the dots is proportional with the number of whines produced. The grey field shows the 95% confidence interval of the linear fit. The significant negative effect remains even after excluding the extreme values (Escape scale > 6).

Dogs that barked and tried to escape from the lab more (higher PC2 scores) during the separation had lower HNR, and thus noisier whines compared to dogs with fewer escape attempts (LM: β =  − 1.657; t =  − 4.969; *p* < 0.001; Fig. 8A,B).

**Figure 9:**
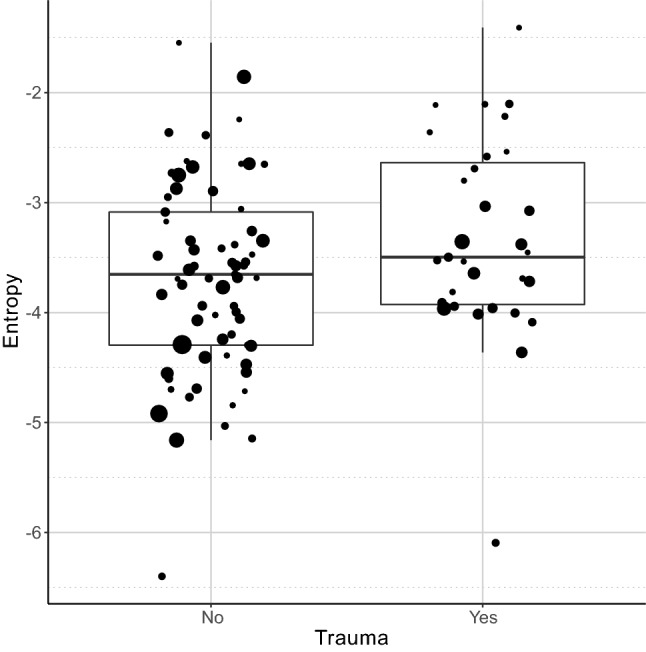
The effect of trauma experienced on the entropy of whines produced during separation. Traumatized dogs had whines with slightly higher entropy. The horizontal line within the box shows the median, the boxes represent the interquartile intervals, the whiskers the range. The size of the dots is proportional with the number of whines produced.

This negative association between escape attempts with tonality (HNR) and higher activity with higher Wiener entropy, both suggest wider spectra and higher noise in the dogs’ whines. As these separation related behaviours are associated with higher negative arousal in dogs (for review see:^38^) possibly due to frustration^45^, our observations are in line with former studies showing that the entropy of contact calls rises with the level of arousal^19–21^. In line with these we found that dogs that experienced traumatic life events and presumably suffer from a higher level of stress when separated from the owner tended to produce whines with higher Wiener entropy (LM: β = 0.352; t = 1.856; *p* = 0.066; Fig. 9), having a slightly more irregular structure and wider spectrum, although this effect was only marginally significant thus should be treated with caution.

### Limitations

Our study has one obvious limitation, the lack of physiological measurements to assess the dogs’ stress level. On one hand we tried to avoid any additional stressor besides the separation itself, thus any invasive measurements (e.g., repeated blood sampling) of stress hormones, or other non-invasive methods like the application of wearable heartrate measuring devices were out of the question. Saliva, urinary or faecal sampling of stress hormones would have been a plausible alternative, but these would have overly lengthened the test procedure. Furthermore, due to the high variability of our sample in size and breed the optimal timing of the sampling would have been hard to determine, especially as our separation was only 3 min long, thus we reasoned that such hormonal measurements would have been too noisy for proper testing. Finally, one option would have been the telemetric measurements of ear temperature like in Riemer et al.^46^ but unfortunately, we had no access to the necessary equipment. However, as several studies already showed that behaviours such as those measured in our test are associated with stress using physiological measurements in separation^47,48^ and in other fearful contexts^49,50^, we can assume that these are reliable proxies of the dogs' stress level in our sample too.

## Conclusion

Our results are the first indications that in contact calls occurrence of NLP are associated with higher negative arousal. In contrast, as the ratio of NLP in these calls were mainly affected by age and reproductive (and ultimately hormonal) status suggests that the prevalence of these irregularities within individuals is mainly affected by the conditions of the vocal apparatus. Finding different associations with different measurements of vocal harshness highlights that as these are the results of different acoustic processes, and thus might be associated with different inner states.

## Methods

### Subjects

We recruited subjects with and without owner-reported SRP through social media and the dog database of the Department of Ethology, Budapest. We directly contacted those owners who reported in the Vocal Dog Questionnaire (https://goo.gl/forms/ahosgzczQN2ajbWt1) that their dogs’ whine in separation contexts and they were willing to participate in ‘live’ experiments.. We sent an online separation questionnaire (see^33^) to these owners, and invited them to participate in our behaviour test. 167 dogs from numerous breeds, (Table S1) participated in the behaviour test. From the 139 whining dogs 18 dogs emitted only whistle whines (*g*_*0*_), thus 121 dogs’ data (which produced lower frequency, harmonic *f*_*0*_ whines) were acoustically analysed (mean age ± SD = 4.57 ± 2.82, N_males_ = 57, N_neutered_ = 73).

### Ethical statement

Owners of the dogs were informed about the goals and circumstances of the experimental procedure a priori and they were present during the tests. We informed them that they could interrupt the experiment and reconsider their participation if—by their judgement—the test was too stressful for the dog. Their informed consent was obtained in written form via filling and signing the Department of Ethology’s standard consent form. The Animal Welfare Committee of the Eötvös Loránd University reviewed and accepted the protocol of the experiment (Ref. no.: PEI/001/1056–4/2015). The tests were performed in accordance with the Hungarian regulations on animal experimentation and the Guidelines for the use of animals in research described by the Association for the Study Animal Behaviour (ASAB) and ARRIVE.

### Separation questionnaire

From the separation questionnaire we collected demographic data about the dogs (age, sex, breed, reproductive status), life history (origin of the dog) coded into traumatic and non-traumatic category (the following were considered as traumatic life events: time spent in shelter, being a stray, had to be rescued from harmful environment), and a yes–no question whether the owner think her/his dog struggles with SRP.

### Separation test set-up

The set-up was based on the protocol of Konok et al. (2011)^51^. The lab (size: 6.27 m × 5.40 m) was empty during the experiment except for one chair for the owner to sit on during the warm-up phase. Prior to the experiment all the tags and other accessories were removed from the dogs’ collar to prevent them from making a clinking noise. During the entire test, the experimenter sat at a computer in a separate room and oversaw the events of the experiment. The computer recorded the six digital cameras’ (Basler sca640-120gc) video stream and the sound from two microphones placed in the room. One, omnidirectional microphone (Sennheiser ME62 with K6 power module) was suspended from the ceiling in the middle of the room to record the ambient sounds, while one shotgun microphone (Sennheiser ME65 with K6 power module) was fixed above the door used by the owner to enter and exit the room to provide a more focused recording of the dogs’ vocalisations. The two microphones’ signals were recorded through a Zoom H4n operating as a USB soundcard on two separate channels synchronized with the video streams, and during the analysis the better signal-to-noise ratio recording was used (in every case the signal of the shotgun microphone).

### Procedure

o **Phase 1:** The owner and the dog on leash entered the lab. The owner sat down on the chair and released the dog. The dog was allowed to move and behave freely. The owner was asked to avoid any interaction with the dog. The phase lasted 1 min.
o **Phase 2**: The owner left the room with minimal interaction with the dog and locked the door. The dog was alone for 3 min.
o **Phase 3:** The owner entered the room, greeted and played with the dog for at least half a minute to release stress.

### Acoustics

The sounds made by the dogs during the separation were recorded as uncompressed PCM wav files (44.1 kHz, 16bit) and analysed using a custom made Praat (versions 6.0 and 6.1)^52^ script. We segmented and annotated these recordings to mark each individual whine containing first fundamental frequency (N = 4086), and omitted the high frequency squeaks (*g*_0_, these secondary fundamental frequencies were present in almost all whining dogs). Then we measured acoustic parameters in these whines (mean HNR, jitter and Wiener entropy), and also marked each whine carrying NLP (frequency jump, subharmonics, deterministic chaos) based on auditory and visual inspection of the calls (reliability was tested on 10% of the sample with Pearson correlation between two independent coders: r = 0.964). Finally, we calculated the ratio of NLP whines by dividing the number of occurrences with the number of all whines containing low fundamental frequency.

### Behaviour analysis

We coded the behaviour including vocalisation types (Table S2) of the dogs using Solomon Coder^53^ (http://solomoncoder.com). We calculated durations of each behaviour during separation (starting with the owner closing the door till opening it upon return).

### Statistics

Analyses were run in R statistical environment^54^ using RStudio^55^. We applied Principal Component Analysis (psych package^56^, principal function with oblimin rotation) to form behavioural scales from the time duration data (excluding whining). The number of extracted principal components (PCs) were determined with parallel analysis (paran package^57^). Five PCs were defined, and their scores were calculated for each individual for further analysis.

To analyse the behavioural scales, we used Linear Models (lm function) with AIC based backwards elimination (drop1 function) to find the parsimonious models. We built separate models to test the effect of individual (SRP + sex + reproductive_status + trauma + age + sex:reproductive_status) and behaviour data (all PCA scales as covariates) on the vocal parameters, normalized with box-cox transformation when necessary. In the case of NLP occurrences, we ran two set of Generalized Linear Models (GLM) (glm function). First, we included all dogs that emitted *f*_0_ whines and the response variable was whether their whines contained NLP coded as a binary variable (binomial GLM). Then in the second analysis, only dogs that emitted *f*_0_ whine with NLP were included and their NLP ratio was used as the response variable in logit link quasibinomial models. We also applied AIC based backwards model selection in these cases.

In the case of the behavioural scales, some dogs had extreme values. Thus, to exclude the possibility of the effect of these suspiciously influential points, all positive findings were also confirmed with the exclusion of these extreme values. In all cases, except the effect of chair/move (PC1) on the occurrences of NLP, significant effects remained after the exclusion of these extreme values, thus we can conclude that the majority of these values are not outliers or influential points but actual extreme cases fitting into the general distribution pattern.

## Supplementary Information

Supplementary Legends.

Supplementary Table 1.

Supplementary Table 2.

Supplementary Table 3.

Supplementary Table 4.

## Data Availability

All data generated or analysed during this study are included in this published article (and its Supplementary Information files).
