# Supplementary material for: Occurrences of non-linear phenomena and vocal harshness in dog whines as indicators of stress and ageing
Source: Sci Rep. 2021 Feb 24;11:4468. doi: 10.1038/s41598-021-83614-1 (PMC7904949; doi:10.1038/s41598-021-83614-1)
Supplement: Supplementary file 1 — Supplementary Legends. [file 41598_2021_83614_MOESM1_ESM.docx]

Occurrences of non-linear phenomena and vocal harshness in dog whines as indicators of stress and ageing

András Marx, Rita Lenkei, Paula Pérez Fraga, Viktória Bakos Enikő Kubinyi & Tamás Faragó

# Supplementary Tables

Table S1: Raw and calculated data, including dogs’ demographic information, behaviour measurements, PCA scores and acoustic measurements

Table S2: The definitions of the coded behaviours

Table S3: The loadings of the extracted Principal Components. Most influential variables’ loadings are highlighted with bold typeface for each PC. Cumulative variance, eigenvalues and standardised alpha values are also reported.

Table S4: The parsimonious models with the retained variable after model selection.
